# Supplementary figures and images for: A highly potent human antibody neutralizing all serotypes of BK polyomavirus
Source: PLoS Pathog. 2025 Jul 18;21(7):e1013122. doi: 10.1371/journal.ppat.1013122 (PMC12289034; doi:10.1371/journal.ppat.1013122)

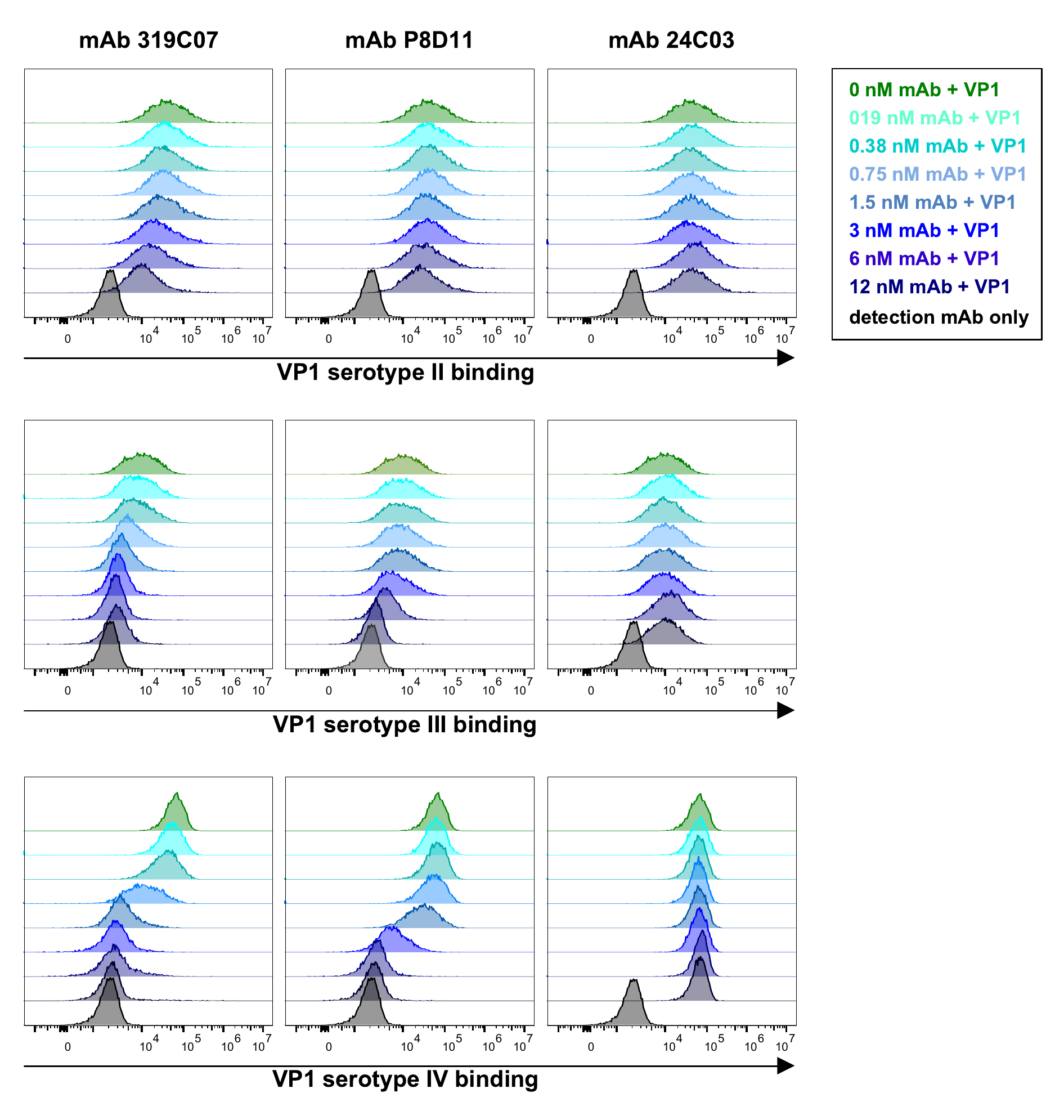

Supplement: S1 Fig — Attachment of VP1 serotypes II, III and IV pentamer to HEK293TT cells in the absence (dark green) or presence of antibody (mAb319C07, mAb P8D11 or mAb 24C03) at different concentrations (shades of blue) was analyzed. VP1 binding to HEK293TT cells was detected using an AF488 labelled anti-VP1 antibody and compared to cells with detection antibody only (black). Analysis was done by analytic flow cytometry. A shift of the HEK293TT population to higher signals (right-shift) over background (black) indicates binding of VP1 to the cells. (TIFF) [file ppat.1013122.s001.tiff]

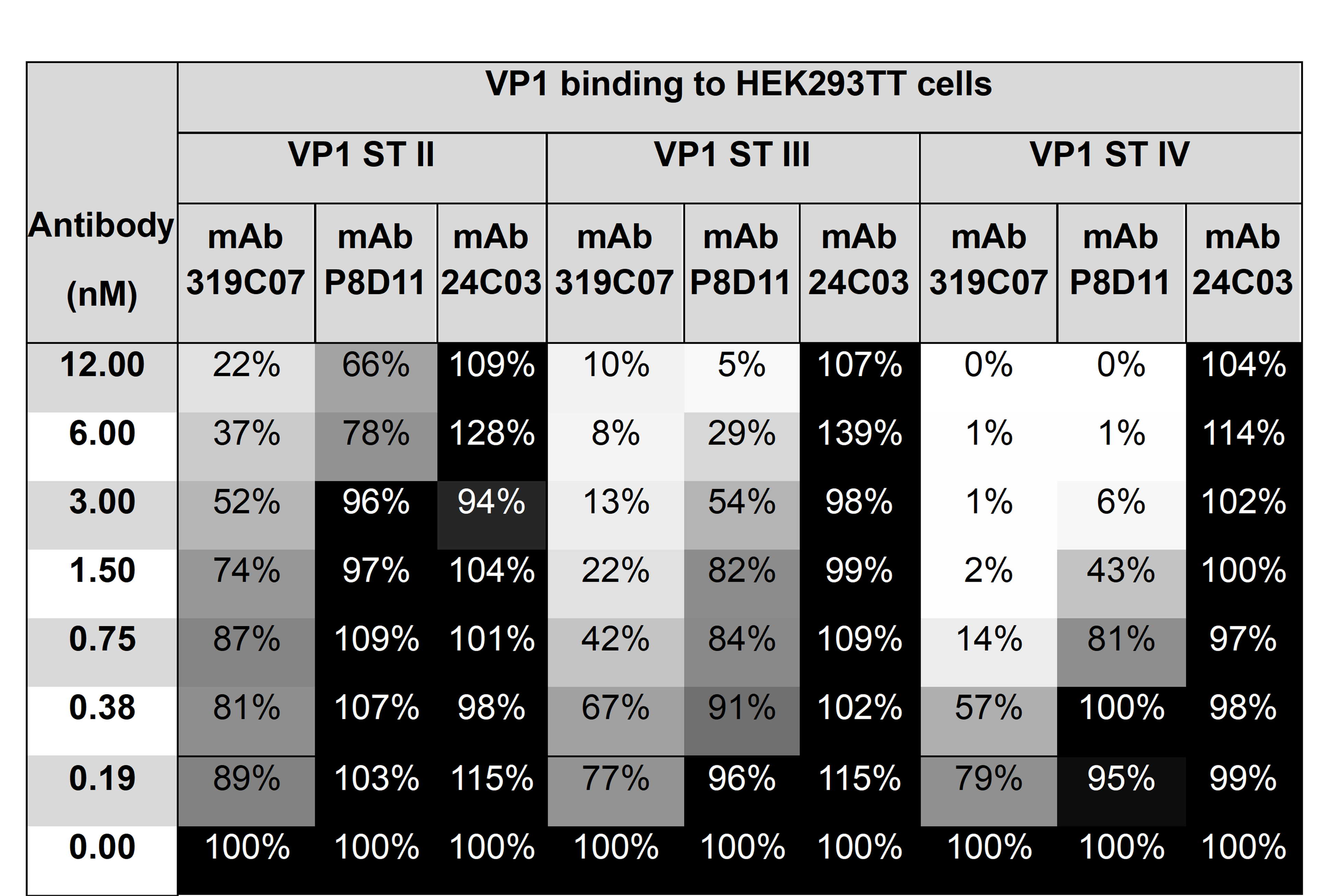

Supplement: S1 Table — Median fluorescent intensity of plots shown in S1 Fig were extracted using FlowJo 10.7.1 and normalized to 100% VP1 binding (VP1 II, III or IV binding to HEK293TT cells in the absence of antibodies) and 0% VP1 binding (HEK293TT cells with detection antibody only). For mAb 319C07 and mAb P8D11, a concentration-dependent decrease in VP1 binding to the cells was observed. mAb P8D11 shows a weaker inhibition of VP1 binding to cells than mAb 319C07 for all VP1 serotypes tested. mAb 24C03 does not inhibit VP1 binding to HEK293TT cells. n = 1, 15000 cells analyzed per concentration. (TIF) [file ppat.1013122.s002.tif]

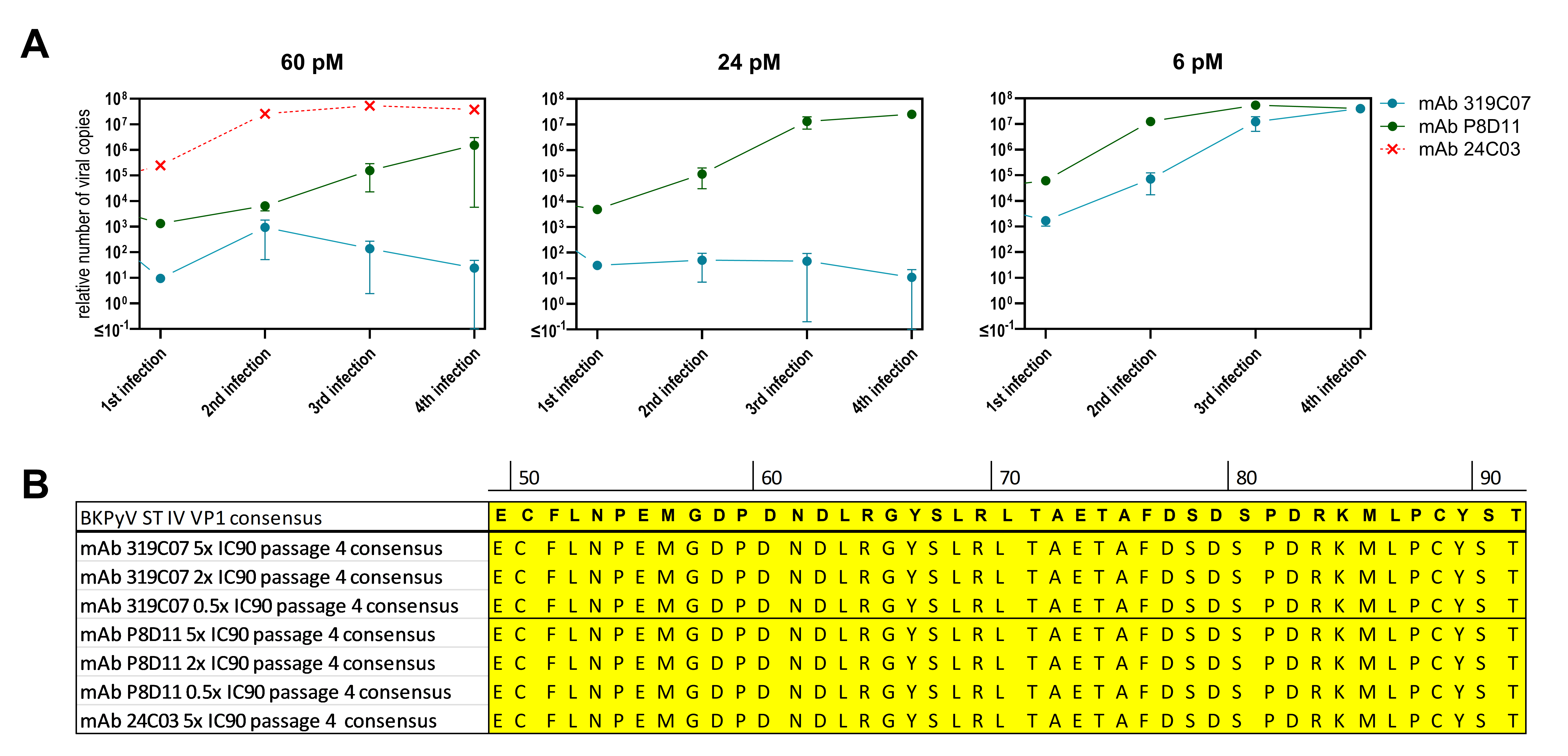

Supplement: S2 Fig — A) Neutralization capacity of mAb 319C07 and reference antibody mAb P8D11 plus negative control mAb 24C03 on HRPTEC infected with BKPyV serotype IV was quantified. With antibodies mAb 319C07 and reference antibody mAb P8D11 assessment was performed in duplicate at all concentrations, for mAb 24C03, the highest concentration was used. Plotted are mean and ranges of n = 2. B) Sequence analysis performed with the 4th infection passage showed no occurrence of mutated virus. (TIF) [file ppat.1013122.s003.tif]
