# Supplementary material for: A highly potent human antibody neutralizing all serotypes of BK polyomavirus
Source: PLoS Pathog. 2025 Jul 18;21(7):e1013122. doi: 10.1371/journal.ppat.1013122 (PMC12289034; doi:10.1371/journal.ppat.1013122)
Supplement: S1 Supplementary Method — (DOCX) [file ppat.1013122.s004.docx]

**Supplementary method**

**Long-term co-culture of replication competent BKPyV STIV on human renal proximal tubular epithelial cells and antibody 319C07**

The long-term neutralizing efficacy of antibody mAb 319C07 against BKPyV serotype IV (generated by introducing the subtyping region of serotype IV into the Dunlop background, see [1]) was assessed as described for BKPyV serotype I with modifications described in the following. Cultivation of HRPTEC was performed in 6-well culture plates, seeding four days in advance to have 200’000 cells/well at assay initiation. At the day of assay start, BKPyV serotype IV at a multiplicity of infection (MOI) 0.1 was mixed with antibody at 9 pg/µl, 3.6 pg/µl and 0.9 pg/µl (5x, 2x or 0.5x IC90 respectively for potravitug) in a total of 3 ml medium per well. The mixture was incubated at room-temperature for 1 h and subsequently used to replace the spent cell medium. To maintain the cells, 0.3 ml medium containing freshly prepared antibodies at the appropriate concentration were added every three days. After 14 days, the viral replication was assessed by qPCR [2] from cell supernatants subjected to viral inactivation by boiling. The assay plate with cells and remaining supernatant underwent freeze-thaw cycles to release mature viral particles for subsequent infection cycles. Of the homogenized mixture, 0.3 ml were mixed with antibodies of the above indicated concentrations and a total volume of 3 ml and incubated at room temperature for 1 h prior to replacing the cell medium of freshly seeded cells for the next culture passage. Reinfection and antibody treatment occurred every two weeks for a total of four cycles.

**References**

1. Tremolada, S.; Delbue, S.; Larocca, S.; Carloni, C.; Elia, F.; Khalili, K.; Gordon, J.; Ferrante, P. Polymorphisms of the BK Virus Subtypes and Their Influence on Viral in Vitro Growth Efficiency. *Virus Research* **2010**, *149*, 190–196, doi:10.1016/j.virusres.2010.01.017.

2. Urayama, T.; Takahashi, K.; Ideno, S.; Yunoki, M.; Saito, M.; Numakura, K.; Inoue, T.; Satoh, S.; Sakai, K. BK Polyomavirus-Neutralizing Activity of Intravenous Immunoglobulin Products Derived from Donated Blood in Japan. *ISBT Science Series* **2016**, *11*, 146–152, doi:10.1111/voxs.12294.
