# Supplementary material for: A highly potent human antibody neutralizing all serotypes of BK polyomavirus
Source: PLoS Pathog. 2025 Jul 18;21(7):e1013122. doi: 10.1371/journal.ppat.1013122 (PMC12289034; doi:10.1371/journal.ppat.1013122)
Supplement: Attachment — Submitted filename: Response to Reviewers.docx [file ppat.1013122.s014.docx]

Response to Reviewers

Manuscript: **A Highly Potent Human Antibody Neutralizing All Serotypes of BK Polyomavirus**

Dear Editors and Reviewers,

We would like to thank you for your thoughtful and constructive feedback on our manuscript. We appreciate the time and effort you have invested in reviewing our work. Below, we address each of the points raised.

**Editor requirements**

Comment #1: Please provide an Author Summary.

Response #1: Author summary added.

Comment #2: We noticed that you used the phrase 'data not shown' in the manuscript.

Response #2: We inserted a new figure into the supplement (Figure S2) showing the previously not included data.

Comment #3: We do not publish any copyright or trademark symbols.

Response #3: Copyright or trademark symbols have been removed throughout the document

Comment #4: We are unable to open the following Supporting Information file: OneDrive_2_14.04.2025.zip. Please kindly revise as necessary and re-upload.

Response#4: As we provide raw data for every figure separately this zip file is not needed any more.

**Reviewer #1**

Comment #5: The new antibody is no doubt highly potent, but in my opinion the manuscript’s writing style (discovery of an “ultra-potent” antibody, etc.) raises expectations that are not quite matched by the data shown in Figure 2 and Table 1. Likewise, the inhibition of binding of BKPyV VP1 to HEK283TT cells (Figure 3) shows a small improvement compared to P8D11.

Response #5: The writing style has been adapted to "highly potent" as the title of the manuscript already suggested.

Comment #6: I found this section (structure) of the manuscript somewhat lacking in detail – it would be helpful I think to include a detailed figure showing the detailed contacts for one Fab, with specific residues. What contacts are formed? Do the interactions preclude binding of (i.e. clash with) the receptor? What is the buried surface area in the complex?

Maybe one could show an overlay of the structures bound to the receptor and to the Fab? I think such a more detailed figure of the interactions would be very helpful.

Response #6: Thank you very much for the comment. We have substantially improved this section (text and figure) and have thoroughly reanalysed the structure, identifying an additional contact of the Fab to a third protomer.

Comment #7: Furthermore, it would also be helpful to highlight residues that differ among the BK genotypes and their role(s) in contact formation, in order to illustrate how the antibody is able to recognize all 4 genotypes. I noted that the antibody 319C07 does not neutralize genotype III nearly as efficiently as the other three genotypes (Table III). Can this be explained by differences in the sequence (specific residues that are different) in the Fab-binding region?

Response #7: We now provide a sequence alignment in Figure 6 D, which highlights differences between serotypes and emphasizes interaction sites of antibody and ganglioside GD3 on VP1. We now also hypothesizes why serotype III is neutralized less efficiently.

Comment #8: I think the manuscript would benefit from the inclusion of affinity data (e.g. surface plasmon resonance), although I don’t see this as a requirement for acceptance.

Response #8: Unfortunately, we are unable to show affinity data by SPR.

Comment #9: Figure 6 needs labels, maybe also highlight one VP1 monomer to show how the Fabs bridge two VP1 monomers.

Response #9: Both comments addressed, labels and highlight added.

Comment #10: The authors write in the discussion that “the joining site between VP1 monomers is highly conserved, and mutations in this region to avoid binding of mAb 319C07 would likely disrupt capsid formation, significantly compromising viral fitness”. I am not sure I follow the reasoning here. I would think that the Fab does not directly bind to residues that are involved in VP1-VP1 interactions within the pentamer, but rather to surface-exposed residues in this area, correct? Thus, mutations of such surface-exposed residues would likely be tolerated by the capsid.

Response #10: We agree that this may be misleading and have rephrased the corresponding part.

**Reviewer #2**

Comment #11: Intro sentence 2: it would be more informative (and less redundant) to say “forms a non-enveloped capsid.”

Response #11: Changed accordingly

Comment #12: Intro sentence 3: BKPyV-IV doesn’t require gangliosides for entry <https://pubmed.ncbi.nlm.nih.gov/23843634/> Instead of “engaging gangliosides in the cell membrane” it would be more accurate to say “engaging sialylated glycans.”

Response #12: Changed accordingly

Comment #13: The last sentence of the first Intro paragraph is missing a reference. The sentence could simply be deleted. If it’s kept, it would also be important to note that bladder epithelial cells are a typical productive target tissue for BKPyV.

Response #13: Reference and note regarding bladder epithelial cells added

Comment #14: Bottom of page 2: cite NCT04294472

Response #14: Added

Comment #15: Top of page 3: consider removing the comma in the phrase “recipients, who” - the comma confusingly seems to imply that all kidney transplant recipients demonstrate fast clearance.

Response #15: Removed

Comment #16: Methods: I’m not finding details on how the VP1 pentamers produced? What was the fluorochrome? Which exact strain was used? It’s puzzling that the manuscript alternates between describing it as Ia and Ib1 because the Ia/Ib1 distinction is based on sequences outside the VP1 ORF (i.e., Ia and Ib1 genotypes are indistinguishable at the level of VP1 protein sequence). Is it possible it was actually a Ib2 strain? This is important because Ib2 has been proposed as a disproportionate culprit behind PVAN in some world regions.

Response #16: We added a section on VP1 pentamer production to the Materials & Methods. Manuscript was also adapted to consistently mention serotype Ib1, which was used throughout the study.

Comment #17: bottom of page 14: typo – “clones cloned”

Response #17: Adjusted

Comment #18: Bottom pf page 17: which BKPyV strain was used for the HRPTEC culture experiment?

Response #18: For the long-term experiment performed in HRPTEC serotype I strain VR-837 was used (info added in materials & methods). The experiment using serotype IV (supplementary info) was based on a plasmid containing the Dunlop genome modified by repacing the subtyping region with that of subtype IV (obtained from S. Delbue). Construction thereof described in Tremolada, S et al. Polymorphisms of the BK virus subtypes and their influence on viral in vitro growth efficiency. Virus Research 210, 149, 190-196, doi: 10.1016/j.virusres.2010.01.017 (reference indicated in the Supplementary methods).

Comment #19: Bottom of page 4: “10,000 IU/mL” could leave readers with the false impression that Infectious Units were measured. It appears that something more like “copies/ml” is being measured. Also note: “viremia” may not be correct – the Hirsch lab recently presented a compelling case for the idea that the measurement would more accurately be described as “DNAemia” https://pubmed.ncbi.nlm.nih.gov/31759262/

Response #19: The text now clarifies that IU stands for international units and was determined by the cobas BKV test by Roche. We also addressed the comment on viral load by replacing by BKPyV-DNAemia throughout the manuscript.

Comment #20: Page 7 (cell attachment results): The statement “These results suggest that the neutralizing effect of both antibodies is likely mediated via blockade of viral attachment to cells” is an over-extrapolation of the data. The results convincingly show that 319C07 prevents the pentamer from engaging sialylated glycans on the cell surface. Although this effect would indeed be neutralizing in many contexts, it’s important to note that the assembled virion also has binding pockets that can engage glycosaminoglycans. https://pubmed.ncbi.nlm.nih.gov/29091757/

The GAG-binding pockets are absent from free pentamers. It thus remains conceivable that a full-size virion could bind the cell surface via engagement of cell-surface GAGs even while 319C07 is blocking the engagement of sialylated glycans. The text should be re-written to focus on the results indicating specific interference with the sialylated glycan-binding step in the infectious entry process, as opposed to all possible forms of cell attachment. There's a similar problem at the top of page 10 – BKPyV has two distinct glycan receptors/co-receptors. It’s important to be specific about the fact that the mAb is blocking the sialylated glycan binding pocket.

Response #20: Thanks for addressing this point. The text is now specifically addressing the points raised, also in the discussion.

Comment #21: Page 11 (long term propagation experiment): since BKPyV is a DNA virus replicated with high fidelity by host cell DNA polymerases, it generally accumulates mutations at a very slow rate. The lack of observed mutations is thus consistent with general theory. On the other hand, it appears that the viral genome can be acutely targeted by host cell APOBEC3B: https://pubmed.ncbi.nlm.nih.gov/29746834/

HRPTEC cells may not express much APOBEC3B in culture. It would be useful for the Discussion section to mention the idea of testing 319C07 against stereotypical APOBEC3B-signature mutations that are typically found in PyVAN cases. It could be framed as a limitation of the current study that might be addressed in future work.

Response #21: We have rephrased the part to clearly indicate that situation in HRPTEC may not reflect situation in patients. On the other hand, we have performed the assay according to the publication by Lindner et al. (https://pubmed.ncbi.nlm.nih.gov/30824324/) who have observed early viral escape in HRPTEC for some antibodies so the mutational mechanisms seem to be working in HRPTEC.

We hope that our revisions and responses adequately address your concerns. Thank you again for your valuable feedback.

Sincerely,

The Authors
